# Overview of Canada’s Antimicrobial Resistance Network (AMRNet): A data-driven One Health approach to antimicrobial resistance surveillance

**DOI:** 10.14745/ccdr.v48i1112a05

**Published:** 2022-11-03

**Authors:** Wallis Rudnick, Shamir N Mukhi, Richard J Reid-Smith, Greg J German, Anil Nichani, Michael R Mulvey, the Canadian Public Health Laboratory Network Antimicrobial Resistance Working Group, the Canadian Animal Health Laboratorians Network Antimicrobial Susceptibility Testing Working Group

**Affiliations:** 1 Science, Reference & Surveillance, National Microbiology Laboratory, Public Health Agency of Canada, Ottawa, ON; 2 Canadian Network for Public Health Intelligence, National Microbiology Laboratory, Public Health Agency of Canada, Edmonton, AB; 3 Centre for Food-borne, Environmental and Zoonotic Infectious Diseases, Public Health Agency of Canada, Guelph, ON; 4 Department of Laboratory Medicine and Pathobiology, University of Toronto, Toronto, ON; 5 Science, Reference & Surveillance, National Microbiology Laboratory, Public Health Agency of Canada, Guelph, ON; 6 Science, Reference & Surveillance, National Microbiology Laboratory, Public Health Agency of Canada, Winnipeg, MB

**Keywords:** surveillance, antimicrobial resistance, antimicrobial susceptibility, one health, bacteria, fungi, Antimicrobial Resistance Network

## Abstract

The Antimicrobial Resistance Network (AMRNet) is a laboratory-based antimicrobial resistance (AMR) surveillance system under development at the Public Health Agency of Canada’s (PHAC’s) National Microbiology Laboratory. The AMRNet surveillance system captures information on antimicrobial susceptibility testing from clinical and veterinary laboratories including both public and private facilities. In the future, the AMRNet system will also capture relevant data from existing PHAC surveillance systems for AMR including the Canadian Integrated Program for Antimicrobial Resistance Surveillance, the Canadian Nosocomial Infection Surveillance Program and the Enhanced Surveillance of Antimicrobial-Resistant Gonorrhea program, and contribute to the Canadian Antimicrobial Resistance Surveillance System. AMRNet’s integrated “One Health” approach will allow health professionals and researchers to take a multi-dimensional perspective of AMR in both human and animal health in Canada and will make Canada a leader in AMR surveillance.

AMRNet is a collaboration between PHAC, provincial and territorial public health organizations as well as clinical and veterinary laboratories across the country. As part of a phased rollout, AMRNet is now collecting human clinical data from three provinces, from both inpatients and outpatients. Ultimately, AMRNet aims to capture all antimicrobial susceptibility testing results from all bacterial and fungal pathogens across Canada.

This article describes the AMRNet surveillance system, including program objectives, system structure and the data collected. The integration of human and animal data in AMRNet will inform One Health responses to AMR issues. The capacity to collect and to disseminate data to stakeholders in real time is a critical step to addressing emerging AMR issues in Canada.

## Introduction

Antimicrobial-resistant organisms are a major global public health concern; the World Health Organization identified antimicrobial resistance (AMR) as a “top-ten” threat to global health in 2019 ((1)). With the increase in antimicrobial-resistant organisms globally and the lack of new antimicrobials in the development pipeline, it is critical that Canada responds to this emerging threat and limits the spread of these organisms to prevent difficult-to-treat infections. Antimicrobial resistant surveillance is critical to Canada’s ability to respond to emerging antimicrobial-resistant organisms and to provide intelligence to limit their spread. The surveillance of AMR was identified as a key pillar in the 2015 *Federal Framework, Antimicrobial Resistance and Use in Canada: A Federal Framework for Action*. This framework outlines the Government of Canada’s commitment to address AMR challenges and the need to expand Canada’s AMR surveillance ((2)).

While Canada has world-class AMR surveillance programs in a variety of settings, there are important gaps in surveillance, notably in the community, long-term care settings and smaller hospitals. Recognizing these gaps, a 2022 evaluation of the One Health AMR surveillance landscape in Canada recommended the “development of a complete, integrated AMR/AMU [antimicrobial use] surveillance program” ((3)). The 2015 Federal Framework describes how the “expansion of community-based surveillance will address a gap in the understanding of antimicrobial resistance” ((2)). The Antimicrobial Resistance Network (AMRNet) surveillance system is designed to address these gaps and to provide a flexible platform that will adapt to emerging and future needs of AMR surveillance in Canada. AMRNet has the potential to expand to include not only new human and animal pathogens but also new domains such as wastewater, AMU and monitoring the susceptibility of newly available or newly commercialized antibiotics in humans and agriculture. Additionally, there is the potential to integrate whole genome sequencing into AMRNet to examine transmission patterns between and within species.

AMRNet will allow for international comparisons and will augment Canada’s contribution to the World Health Organization Global Antimicrobial Resistance and Use Surveillance System. Large laboratory-based AMR surveillance systems have been developed internationally including the European Antimicrobial Resistance Surveillance Network ((4)), the United States Centers for Disease Control and Prevention’s Antimicrobial Resistance Laboratory Network (AR Lab Network) and the Global Antimicrobial Resistance Laboratory and Response Network ((5)).

Over the years, many Canadian jurisdictions have made significant strides towards capturing and standardizing lab-based AMR data and AMU data in their jurisdictions ((6–14)). The scope and design of these programs vary, but all have increased the availability of AMR-related data in Canada. These achievements have laid the groundwork for the development of a Canada-wide system for AMR-related data.

The Public Health Agency of Canada (PHAC) has long-standing programs for capturing data on AMR in various settings, including the Canadian Nosocomial Infection Surveillance Program (CNISP), the Enhanced Surveillance of Antimicrobial-Resistant Gonorrhea (ESAG) and the Canadian Integrated Program for Antimicrobial Resistance Surveillance (CIPARS). AMRNet will work with federal partners to incorporate data from these programs to fill gaps in AMR data that would not be otherwise collected by front line laboratories and for in-depth investigations of AMR issues when identified.

## Description of the AMRNet surveillance system

AMRNet is a collaboration between PHAC, provincial and territorial public health organizations as well as clinical and veterinary laboratories across the country. The AMRNet surveillance system captures information on antimicrobial susceptibility testing from laboratory information systems in clinical and veterinary laboratories, including both public and private facilities, and including reference laboratories. The AMRNet system will also capture data from long-standing PHAC surveillance programs that conduct in-depth AMR surveillance in specific settings (e.g. CNISP, ESAG and CIPARS). Ultimately, AMRNet aims to capture all antimicrobial susceptibility testing results from all bacterial and fungal pathogens across Canada.

Objectives of the AMRNet surveillance program include the following: 1) to integrate monitoring of trends in AMR rates across human and animal populations, nationally, regionally and locally; 2) to detect emergence and spread of AMR in Canada; 3) to disseminate timely information on AMR in Canada; 4) to fulfill Canada’s commitment to the World Health Organization’s Global Antimicrobial Resistance and Use Surveillance System initiative; 5) to support research and innovation on AMR; and 6) to build antimicrobial stewardship capacity at provincial/territorial/local public health levels.

**Figure 1:**
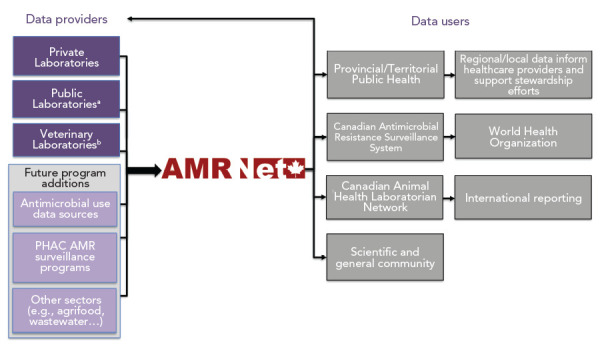
Data flow for AMRNet surveillance system Abbreviations: AMR, antimicrobial resistance; AMRNet, Antimicrobial Resistance Network; PHAC, Public Health Agency of Canada ^a^ Laboratories funded, managed or operated by governmental health organizations ^b^ AMRNet is currently conducting surveillance among veterinary laboratories as a pilot program

To meet these objectives, the AMRNet team has worked closely with the Canadian Public Health Laboratory Network’s (CPHLN) AMR Working Group to ensure provincial and territorial AMR needs are met. AMRNet collects antimicrobial susceptibility testing results of bacterial and fungal pathogens, along with select patient or animal characteristics (**Table 1** and **Table 2**). These “linelist” data are captured from laboratory information systems in clinical and veterinary laboratories (**Figure 1**). Ideally, AMRNet captures both the minimum inhibitory concentration (MIC) value and the interpretation (e.g. susceptible, intermediate, resistant) of each result. Currently, capturing MIC values is not feasible for all jurisdictions and thus MIC values are not a mandatory field for data submission.

**Figure 2:**
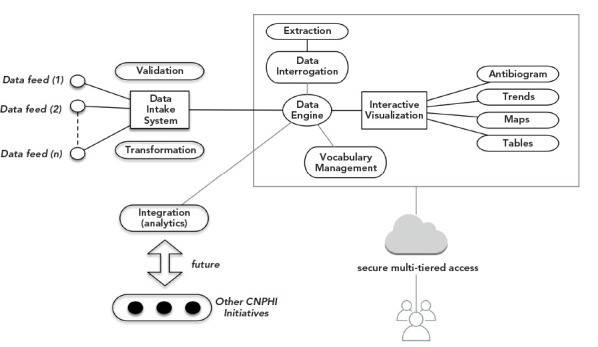
High-level depiction of the technical vision behind the AMRNet initiative on the Canadian Network for Public Health Intelligence platform Abbreviations: AMRNet, Antimicrobial Resistance Network; CNPHI, Canadian Network for Public Health Intelligence

**Table 1:** Mandatory and optional data elements for AMRNet surveillance among humans

Category	Data elements
Mandatory	Unique patient identifier Age group^a^ Sex Forward sortation area^a^ or region^b^ Inpatient versus outpatient Date of isolation or collection Specimen identifier Organism (genus and species) Interpretation (susceptible, intermediate, or resistant) results for each antimicrobial Source/anatomical site of culture Data source/submitting organization Province/region of data submitter
Optional	Minimum inhibitory concentration results Nosocomial acquisition/hospital origin Patient setting details (e.g. ward, clinic, etc.) Subtype/serotype of bacteria/fungi Laboratory comments Other data elements selected by the data provider

Abbreviation: AMRNet, Antimicrobial Resistance Network

^a^ Forward sortation area: first three digits of postal code

^b^ Granularity of data collection determined by population size and privacy considerations in the jurisdiction

**Table 2:** Mandatory and optional data elements for AMRNet pilot programs among animals

Category	Data elements
Mandatory	Unique submission identifier Animal species Province where animal lives or veterinary clinic operates Pooled vs individual animal Date of isolation or collection Specimen identifier Bacteria genus/species Interpretation (susceptible, intermediate, or resistant) results for each antimicrobial Source/anatomical site of culture Data source/submitting organization
Optional	Duplicate specimens identified Screening specimens identified Minimum inhibitory concentration results Additional animal characteristics (e.g. age, commodity, etc.) Subtype/serotype of bacteria/fungi Locality where animal lives^a^ Specimen comments

Abbreviation: AMRNet, Antimicrobial Resistance Network

^a^ Granularity of data collection determined by population size and privacy considerations in the jurisdiction

The data captured by AMRNet will be used to understand trends in AMR at the national and regional level, to identify areas for in-depth investigations and to fulfill Canada’s obligations for international reporting. These data will provide Canadians with tools to better understand AMR trends from a One Health perspective in Canada and around the world. Data from AMRNet will also enable subgroup analyses by sex and age group.

Through standardization and automation, AMRNet aims to make AMR data and analyses more timely and accessible for the organizations submitting AMR data. Once data have been submitted and validated, data providers will be able to download their cleaned and standardized data. Data providers will also be able to explore their data through the creation of antibiograms and visualizations within the AMRNet module. In addition to viewing their own data, data providers can compare their data to other regions in their province, to other regions in Canada (i.e. West, Central, East), and to national data. It will also be possible to monitor trends in multidrug-resistant organisms or extensively drug-resistant organisms following Canadian recommendations on laboratory interpretation ((15)).

### Inclusion and exclusion criteria

For specimens from humans, AMRNet collects data on all antimicrobial susceptibility results from bacterial/fungal pathogens regardless of whether the results were reported to physicians. Duplicate specimens from the same patient are identified or removed as per the recommendations of the Clinical and Laboratory Standards Institute ((16)). Screening specimens are also identified or removed prior to submission.

These data are extracted from laboratory information systems using existing or newly developed procedures and subsequently uploaded to a secure online AMRNet system (see **Figure 2** for description of the AMRNet system on the Canadian Network for Public Health Intelligence, CNPHI). Data may be transferred daily through an automated process or less frequently if automation is not feasible for the data provider. Once the data are uploaded and validated, the standardized data will be available for data providers to access and download. Data providers will be able to access their own data as well as aggregate data from other providers. Antibiograms and data visualizations are under development.

### Annual collection of metadata

In addition to the line-list data, the AMRNet surveillance system will collect metadata from each data provider. These metadata will inform data interpretation and improve understanding of system limitations. These data will be collected via an annual questionnaire and will include the following: geography and time period covered by the data submission; specimen types, organisms and antimicrobials included in the data submission; breakpoint interpretations used; details of relevant testing cascades; and laboratory methods (e.g. type of panels, software versions, specialized testing).

### Animal surveillance pilot program

Three pilot projects are underway to capture data from veterinary laboratories in three provinces. There are additional challenges implementing surveillance among veterinary laboratories as veterinary breakpoints to indicate susceptibility or resistance are not always available ((17)), and procedures for producing antibiogram data are less standardized between laboratories. In addition, there is variation in what data elements are captured electronically. The feasibility of collecting the proposed data elements, de-duplication strategies and the identification of screening specimens are under evaluation in these pilot projects. AMRNet has engaged with the Canadian Animal Health Laboratorians Network Antimicrobial Susceptibility Testing (CAHLN AST) Working Group to seek advice and recommendations on these challenges.

### Role of the Canadian Network for Public Health Intelligence

The CNPHI, an initiative of the National Microbiology Laboratory, is a secure platform of purpose-built technology resources designed to support and enable Canada’s national public health community.

The CNPHI works closely with multi-jurisdictional program partners to provide agile and innovative scientific public health informatics solutions and progressively enhance disease surveillance, preparedness and response capabilities, while fostering intelligence generation and the advancement of research.

Recognizing the importance of AMR as a public health issue, CNPHI played an early role in discussions with partners involved in AMR-related surveillance, fostering collaborative participation and a technical vision for bringing together various initiatives and data streams into a broader, unified picture.

With wide agreement that AMR surveillance is best optimized through an integrated (One Health) approach, CNPHI is proud to contribute as the technical lead, working in close partnership with the experts at the AMRNet Program to help enable the tools and capabilities that can bring a broad AMR surveillance picture into focus.

## What is next?

### Governance

The AMRNet is a collaborative effort between PHAC, provincial and territorial public health and clinical and veterinary laboratories. The AMRNet is engaged with the CPHLN AMR Working Group as well as the CAHLN AST Working Group to provide recommendations and guidance on the development of the human and veterinary programs, respectively.

An initial governance structure is being formalized. An AMRNet Working Group will be responsible for overseeing program development and direction. It will include representatives from PHAC programs as well as from AMRNet advisory groups. The AMRNet Working Group will create advisory groups to provide expertise, consultation and recommendations across various domains. The advisory groups will include representation from the following: provincial and territorial laboratories; CPHLN and CAHLN; federal partners; data users (including clinicians, veterinarians and pharmacists); and other stakeholders.

Advisory groups will include groups for human surveillance, animal surveillance, data privacy and ethics, as well as data access. Advisory groups can be permanent or time limited.

### Cross country rollout

After starting as a series of pilot projects, AMRNet began collecting routine data from a subset of provinces in 2022. Currently AMRNet is collecting data from approximately 1.5 million bacterial and fungal isolates per year from Ontario, Saskatchewan and Prince Edward Island (duplicate isolates excluded as per recommendations of the Clinical and Laboratory Standards Institute) ((16)). The first publication of AMRNet data from these jurisdictions will be included in the *Canadian Antimicrobial Resistance Surveillance System Report* in November 2022 ((18)).

From discussions with provincial and territorial representatives, it is clear that ease of participation will vary by jurisdiction, but AMRNet is slated to rollout across the country in the coming years. PHAC will work with provinces and territories on developing agreements and building the technical capacity for data sharing.

Although AMRNet aims to collect line list data for all requested variables from all bacterial and fungal susceptibility results, this is currently challenging in some jurisdictions due to technical difficulties, resource limitations or other structural barriers. In these situations, AMRNet will work with jurisdictions to build capacity and work towards full program participation. In the short-term, submission of only priority organisms ((19)), exclusion of some variables or data aggregation may be feasible interim solutions. Differences in methods, reporting processes and data availability across jurisdictions will present challenges in interpreting these data.

PHAC will work with partners on data validation and interpretation to ensure integrity of presented data. AMRNet will form but one component of PHAC’s AMR surveillance. As a lab-based surveillance program, AMRNet will conduct wide-scoping surveillance across all bacterial and fungal organisms but will collect limited epidemiological data and will not collect isolates. Other surveillance programs focus more narrowly on particular organisms or infection types but collect detailed epidemiological information and often include the collection of isolates. While AMRNet will be well poised to identify emerging issues, surveillance programs such as CNISP, ESAG and CIPARS will be better suited for in-depth epidemiological investigations.

## Conclusion

AMRNet is a unique collaboration that will provide valuable information on existing and emerging AMR in Canada and help fulfill Canada’s international commitments. The capture of susceptibility testing results from all settings and patient types will close gaps in the Canadian AMR surveillance landscape. The integration of human and animal data will inform One Health responses to AMR issues. The capacity to collect and to disseminate data to stakeholders in real time is a critical step in helping Canadian health professionals detect and respond to emerging AMR issues.
